# Supplementary material for: Evolutionary dynamics of whole-body regeneration across planarian flatworms
Source: Nat Ecol Evol. 2023 Oct 19;7(12):2108–24. doi: 10.1038/s41559-023-02221-7 (PMC10697840; doi:10.1038/s41559-023-02221-7)
Supplement: Supplementary file 1 — Supplementary Figs. 1–6, Tables 3–5 and captions for Tables 1, 2 and 6. [file 41559_2023_2221_MOESM1_ESM.pdf]

nature ecology & evolution

Article

https://doi.org/10.1038/s41559-023-02221-7

Evolutionary dynamics of whole-body regeneration across planarian flatworms

In the format provided by the authors and unedited

Supplementary figures

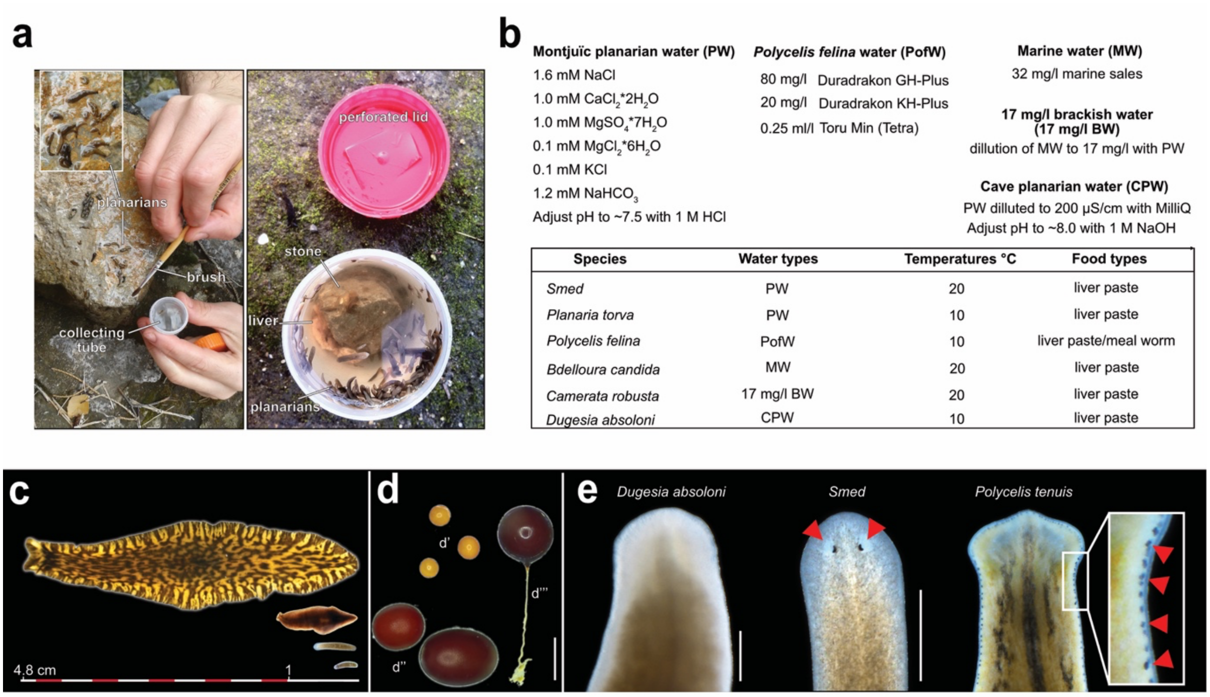

Supplementary Figure 1. a, Illustration of the two predominant planarian field sampling strategies. Manual sampling with brushes (left) or simple, baited traps (e.g., liver) (right). **b,** Formulation of the four main culture media (water types) currently in use in our species collection. The indicated components are diluted with autoclaved Milli Q water unless otherwise indicated. pH is only adjusted for “Montjuïc planarian water”. Below: representative examples of species-specific culture conditions (water type, temperature and food types). **c,** Live image montage illustrating the dramatic body size variations in different planarian species. From top to bottom: *Bdellocephala angarensis*, *Bdellocephala brunnea*, *Smed* (asexual strain) and *Camerata robusta*. **d,** Live image montage illustrating the large differences in egg capsule size and shape between different species. d’ *Camerata robusta*; d’’ *Polycelis tenuis*; d’’’ *Smed*. **e,** Live image montage illustrating the variability in eye number and placement between different species. Red triangle, eyes. Scale bar: 1 mm unless noted.

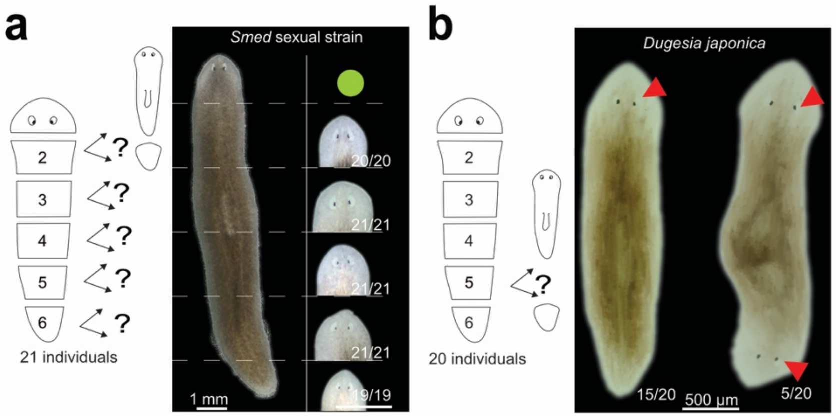

Supplementary Figure 2. a, Cartoon representation of the amputation assay and live images documenting robust head regeneration in *Smed* sexual strain individuals. The two *Smed* strains are indistinguishable by this assay (compare to Fig. 2a). **b,** regeneration of double-headed animals from posterior pieces in *Dugesia japonica*. The relative frequency (observed/total) of double head regeneration for piece 5 is indicated. Red triangles: eyes as morphological markers of head regeneration.

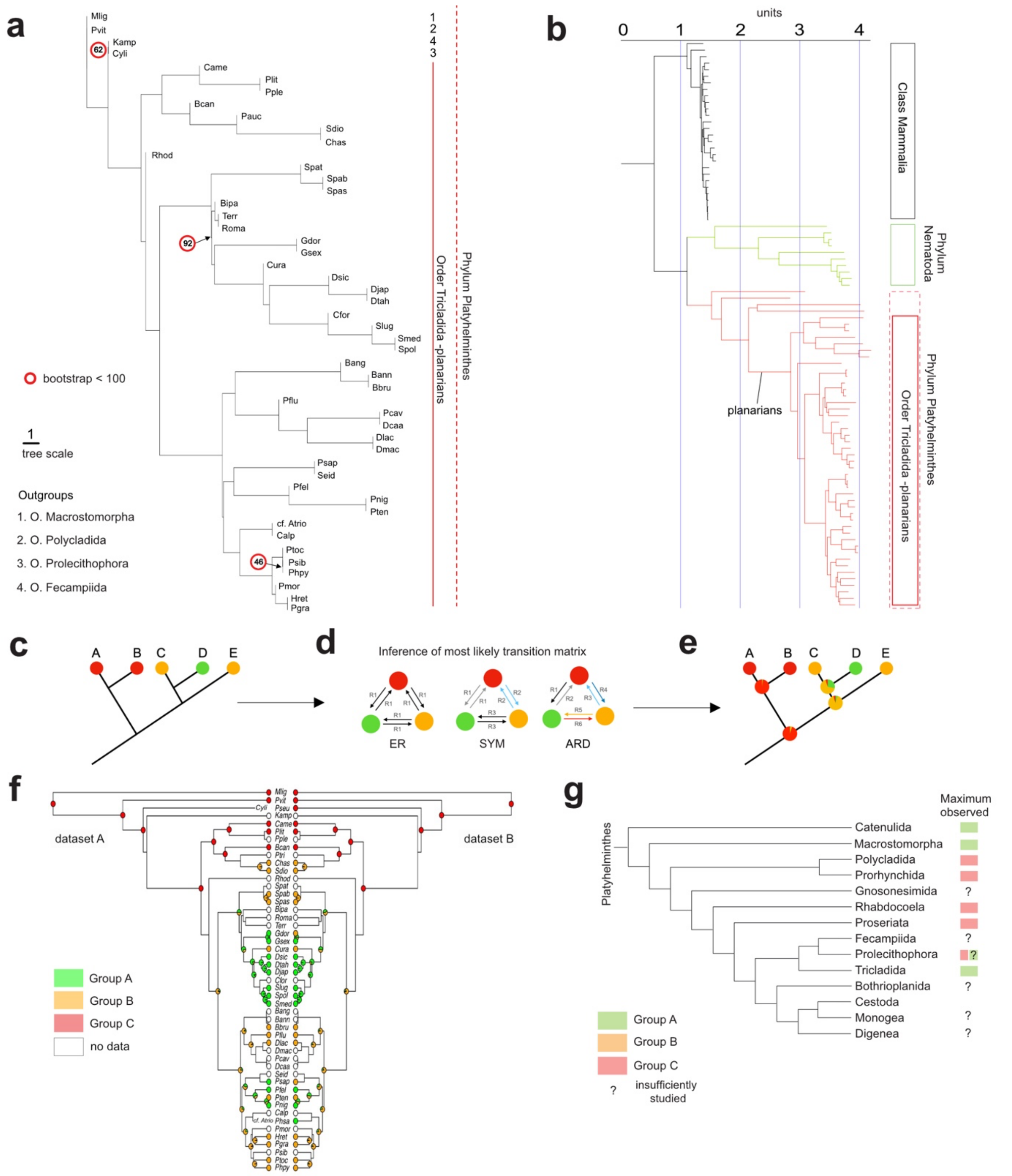

Supplementary Figure 3. a, Astral tree inferred from our dataset with representatives of all the suborders of the Tricladida. The tree topology is similar to that of the Maximum-Likelihood (ML) tree in Fig. 3d, thus further supporting our underlying phylogenetic hypotheses. **b**, Quantitative branch length comparison between mammals, nematodes and Platyhelminthes via Maximum-Likelihood (ML) analysis (see methods). The branch lengths of planarian clades and species (red) indicate much greater sequence divergence than amongst mammals (black) and en par with the sequence divergence amongst the indicated nematode species (green). **c-e**, Schematic workflow of the ancestral character state reconstruction analysis. **c,** Character states found in extant species are used as the input for the analysis. **d,** Given the tip states from c**,** the most likely transition probability

between character states is determined through model comparison. **e**, Stochastic character mapping is used to sample character histories from the most likely model in d. **f**, Inference of ancestral state reconstruction (see methods). We created two species datasets to control for the species with population-dependent variation in head regeneration ability, *Girardia dorotocephala* and *Polycelis sapporo*. Those two species are categorised in group A in dataset A and in group B in dataset B. Figure 3e shows the analysis for dataset A. Data summarised in Fig. 3e corresponds to dataset A. Pie charts at nodes show the proportion of character histories with the indicated state for the capability to regenerate. **g**, Head regeneration capacity in all major free-living Platyhelminthes taxa, modified from[1,2] and adapted to our head regeneration categories.

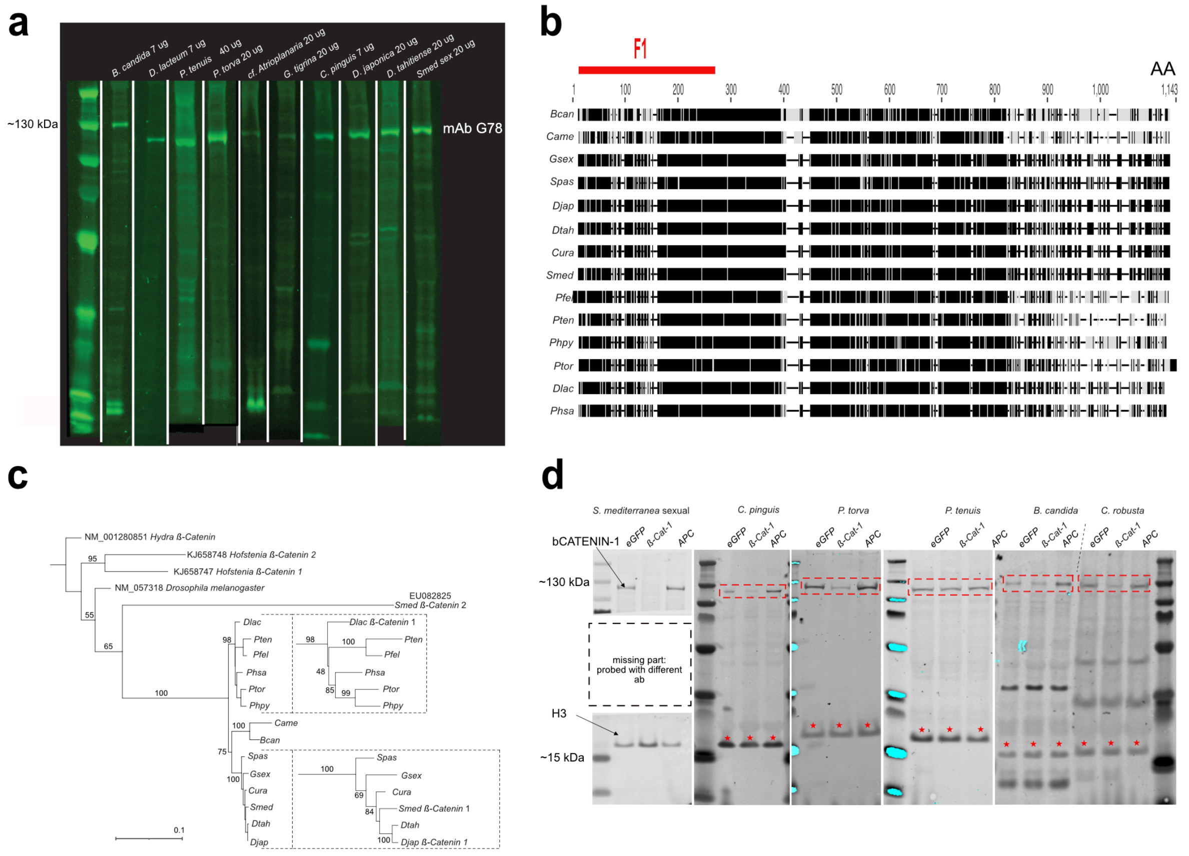

Supplementary Figure 4. a, Uncropped version of the fluorometric Western blot gel image shown in Fig. 4a, displaying the G78 anti-*ß*-CATENIN-1 antibody specificity and background reactivity in different planarian species. Left lane: Molecular weight markers. mAb G78: position of the likely *ß*-CATENIN-1-band in the different species. **b,** Sequence alignments of the *Smed ß*-CATENIN-1 protein sequence with the *ß*-CATENIN-1 homologues of the 13 planarian species for which the species-specific G78 affinity was calibrated. Black bars: Sequence conservation. Red line: The N-terminal fragment of *Smed ß*-CATENIN-1 that was used as antigen for G78 mAb production. **c,** Phylogenetic analysis of the *ß*-CATENIN-1 orthologues from different planarian species. ML tree inferred from published *ß-Catenin-1* gene sequences (*Smed*, *D. lacteum*, *D. japonica*) and other *ß*-CATENIN-1 orthologues recovered by reciprocal BLAST with *Smed ß*-CATENIN-1. Dashed insets: zoom of selected branches, mirroring the phylogenetic distance between the species. **d,** Uncropped version of the fluorometric Western blot gel image shown in Fig. 4b, probed simultaneously with the G78 anti-*ß*-CATENIN-1 mAb and Histone H3 ab (loading control). The image shown is a monochrome-merge of the two separate antibody channels. Planarian species and *RNAi*-conditions as indicated. Molecular weight markers: Left-most lane for each species block, or right-most lane for the *B. candida*/*C. robusta* block. Red frames: quantified *ß*-CATENIN-1 bands, red stars: quantified H3 bands. Note that signal saturation (turquoise overlay) is limited to the molecular weight markers that were not analysed quantitatively.

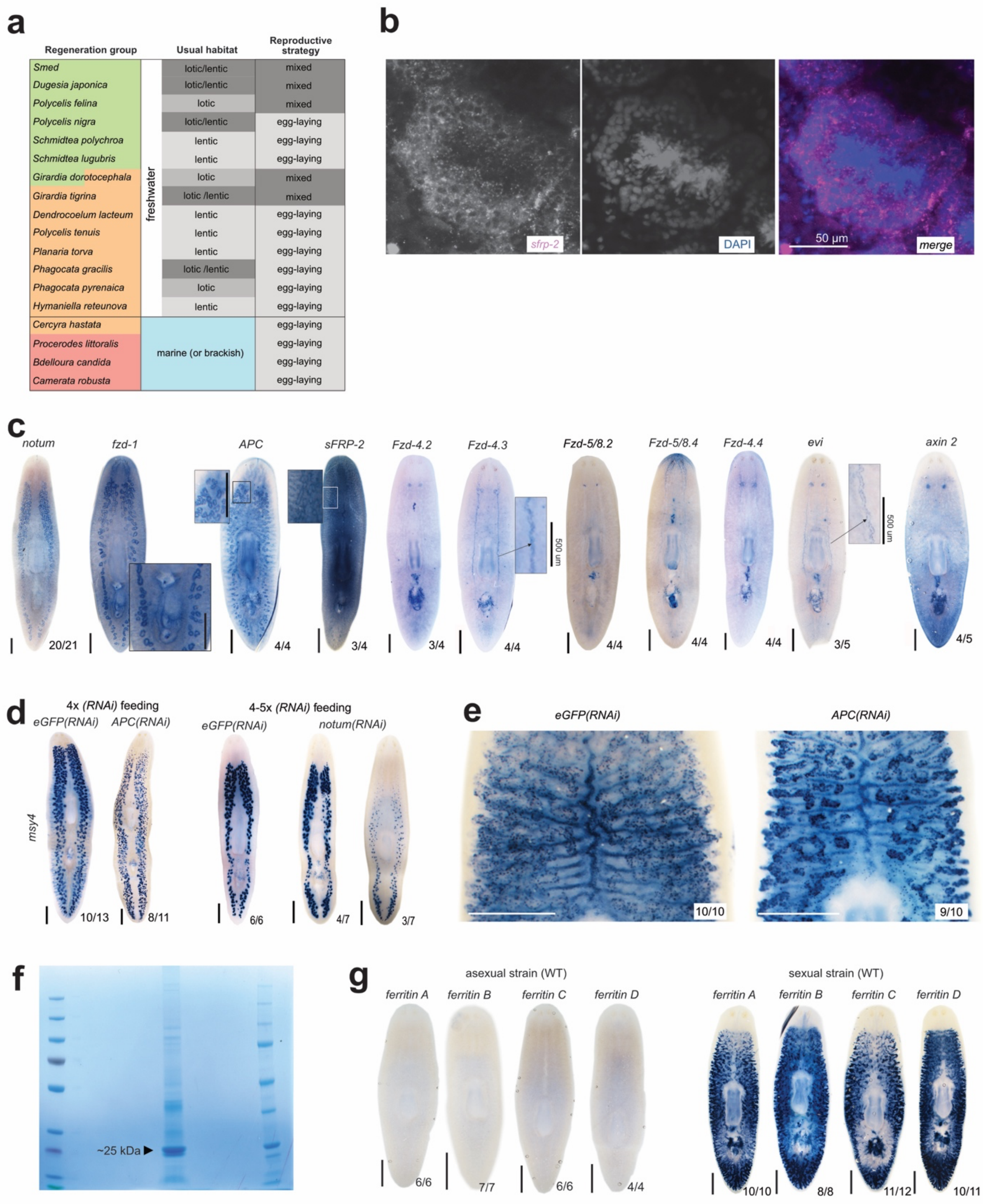

Supplementary Figure 5. a, Correlation between head regeneration capacity, habitat and reproductive strategy in selected planarian species. Lentic: standing freshwater habitats (e.g., ponds, lakes, vernal pools). Lotic: flowing freshwater habitats (e.g., creeks, rivers, springs). The “mixed” reproductive strategy designation indicates that both egg-laying and fission are known for the species. **b,** Fluorescent situ hybridisation expression pattern of *sfrp-2* in a *Smed* testes lobule. Nuclear counterstain: DAPI. **c,** Whole-mount colorimetric (NBT/BCIP) in situ hybridisation expression patterns of selected Wnt signalling pathway genes in the reproductive system of sexual *Smed* individuals. Gene names as indicated. **d,** Whole-mount colorimetric in situ hybridisation expression patterns of the

testes marker *msy4* after *eGFP, APC(RNAi) (left)* and *eGFP*, *notum(RNAi) (right)*. **e,** colorimetric whole-mount in situ hybridisation (detail of Fig. 5f), illustrating denser or clumped staining of the yolk marker *surfactant b* in *APC(RNAi)* as compared to control *(eGFP(RNAi)) specimens*. **f,** SDS-page gel analysis of a *Smed* sexual strain extract (central lane), illustrating the two bands analysed via mass spectrometry (black arrowhead). Molecular weight markers to the left and right. **g,** Whole-mount colorimetric in situ hybridisation expression patterns of the indicated four yolk *ferritin*s in asexual (left) and sexual (right) S*med.* Scale bar: 1 mm unless otherwise noted; number pairs indicate the relative frequency of the shown pattern.

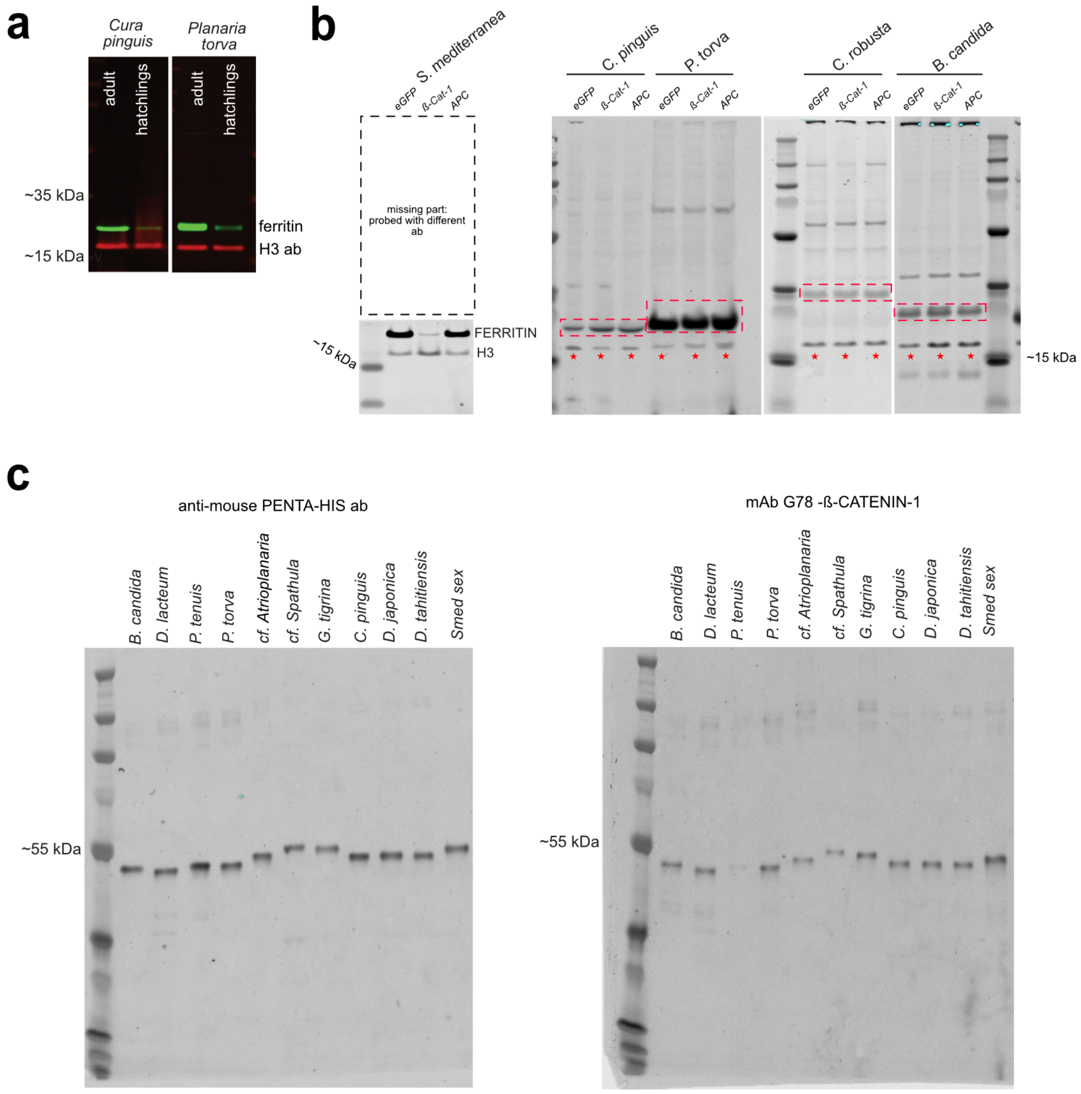

Supplementary Figure 6. a, Fluorometric Western blot probed with the anti-FERRITIN mAb clone EO95 (green) and histone H3 (red) of protein extracted from *C. pinguis* and *P. torva*. The much higher band intensity in sexually mature adults as compared to immature hatchlings supports the utility of EO59 as yolk marker in other species. **b,** Uncropped version of the fluorometric Western blot gel image shown in Fig. 6b, probed simultaneously with E95 mAb (anti-FERRITIN) and H3 (loading control). The image shown is a monochrome-merge of the two separate antibody channels. Planarian species and *RNAi*-conditions as indicated. Molecular weight markers: Left-most lane for each species, but right-most lane for *B. candida*. Red frames: quantified *ß*-CATENIN-1 bands, red stars: quantified H3 bands. **c,** Uncropped versions of the fluorometric Western blot gel image shown in Fig. 6g. Recombinant His-tagged *ß*-CATENIN-1 fragments of the indicated species were run out on a polyacrylamide gel and transferred to a membrane, which was probed simultaneously with anti penta-His (left) and G78 mAb (right). Left-most lane: Molecular weight marker.

References

1 Egger, B., Gschwentner, R. & Rieger, R. Free-living flatworms under the knife: past and present. *Dev. Genes Evol.* **217**, 89-104, doi:10.1007/s00427-006-0120-5 (2007).

2 Grosbusch, A. L., Bertemes, P., Kauffmann, B., Gotsis, C. & Egger, B. Do not lose your head over the unequal regeneration capacity in prolecithophoran flatworms. *Biology* **11**, doi:10.3390/biology11111588 (2022).

Supplementary tables

Supplementary Table 1 (separate Excel file). Head regeneration abilities of planarian strains or species analysed in this manuscript. For some species, more than one field isolate was analysed (e.g., three asexual isolates of *Dugesia sicula* showing similar head regeneration ability). The names of outgroup species are in bold. ?*: insufficiently studied species, but with reported indications of robust head regeneration abilities (e.g., reports of asexual reproduction or head regeneration analysis with cutting paradigms different from the ones used in this paper). n/a: not analysed.

Supplementary Table 2 (separate Excel file). Presence (“1”, green cell) or absence (“0”, white cell) matrix of individual BUSCO genes (Y-axis) in the flatworm transcriptomes (X-axis, in phylogenetic order) (red frame in Fig. 3c), absent from > 90% of the transcriptomes. Red cells: BUSCO genes absent from all transcriptomes. Values for *S. mediterranea* are highlighted in blue for reference.

	dfs	Log.lik	*AICc*	*AIC*	*⊿AICc*	*AICc*weight
**Dataset A**						
ER	1	-25.8	53.6	53.7	2.0	0.3
SYM	3	-22.3	51.6	50.7	0.0	0.7
ARD	6	-22.0	59.5	56.0	7.9	0.0
**Dataset B**						
ER	1	-28.9	59.9	60.0	2.6	0.2
SYM	3	-25.2	57.2	56.4	0.0	0.8
ARD	6	-24.2	63.9	60.4	6.6	0.0

Supplementary Table 3. Model comparison for species datasets A and B. To control for the species with population-dependent variation in head regeneration ability, *Girardia dorotocephala* and *Polycelis sapporo*, we created two species datasets. Those two species are categorised in group A in dataset A and in group B in dataset B. The symmetric model is the preferred model for both datasets.

Dataset A

Model	Good > Medium	Medium > Good	Medium > Bad	Bad > Medium	Good > Bad	Bad > Good	Total
ER	5.3	5.4	4.4	3.3	3.9	2.8	25.3
SYM	4.5	5.9	0.6	2.2	0.0	0.0	13.2
ARD	4.5	5.8	0.0	2.1	0.0	0.0	12.4
**Dataset B**							
ER	4.9	6.3	4.7	3.4	3.8	2.6	25.8
SYM	5.2	6.3	0.5	2.2	0.0	0.0	14.2
ARD	6.7	7.1	0.0	2.1	0.0	0.0	15.8

Supplementary Table 4. Summaries of the number of transitions in stochastic character mappings of datasets A and B (see methods for details on dataset characteristics). Given is the average number of transitions between states across 10.000 simulated character histories. The symmetric (SYM) model was preferred in both datasets (see S. Table 2) and is highlighted in grey. Data is used in Fig. 3e and S. Fig. 3f. The number of transitions is higher than those plotted in Fig. 3e and S. Fig. 3f figures since intra-branch transitions computed in the table are not plotted in the figure.

a) Effect of regneration ability on *ß-CATENIN-1* abundance

Predictors	Estimates	CI	p	N species	Observations	Marginal R2	Conditional R2
(Intercept)	1.72	1.55 – 1.88	<0.001	13	37	0.121	0.762
regeneration category A	-0.11	-0.27 – 0.06	0.176				

b) Effect of sexual system on *ß- CATENIN -1* abundance

Predictors	Estimates	CI	p	N species	Observations	Marginal R2	Conditional R2
(Intercept)	1.69	1.52 – 1.87	<0.001	12	34	0.179	0.764
Asex	-0.14	-0.31 – 0.04	0.106				

c) Effect of regneration ability on yolk abundance

Predictors	Estimates	CI	p	N species	Observations	Marginal R2	Conditional R2
(Intercept)	1.3	1.19 – 1.41	<0.001	9	29	0.244	0.377
regeneration category A	-0.12	-0.22 – -0.01	**0.039**				

Supplementary Table 5: Linear mixed model analyses outcomes for a) Regeneration capability vs *ß-Catenin-1* abundance, b) Sexual system vs *ß-Catenin-1* abundance, and c) Regeneration capability vs yolk abundance. The legend presents the predictors, estimated effect size, confidence interval, p-value, species count in the analysis, total observations, variance proportion attributable to the predictor, and the total model variance inclusive of the random effect.

Supplementary Table 6 (separate Excel file). Primer and gene sequences used in this study. GeneBank accession IDs are listed for all the sequences. Note that only the primer sequences for newly cloned genes are listed. Constructs 1 to 14: ß-Catenin-1 N-terminal fragments of multiple species for the assessment of relative mAb affinities. Gene fragments were cloned into pAff8c and used as template for in-vitro translation. For constructs 40 to 44, the full length ß-Catenin-1 cDNA was first cloned into the pPRT4P vector, which was then used as template for amplifying shorter fragments for dsRNA production (last two primer columns). Vector references for the pPRT-4P and the pAff8c vectors are listed at the bottom of the table.
