# Supplementary material for: Evolutionary dynamics of whole-body regeneration across planarian flatworms
Source: Nat Ecol Evol. 2023 Oct 19;7(12):2108–24. doi: 10.1038/s41559-023-02221-7 (PMC10697840; doi:10.1038/s41559-023-02221-7)
Supplement: Supplementary file 2 — Reporting Summary [file 41559_2023_2221_MOESM2_ESM.pdf]

nature portfolio

Corresponding author(s): Miquel Vila Farré
Jochen C. Rink

Last updated by author(s): May 15, 2023

Reporting Summary

Nature Portfolio wishes to improve the reproducibility of the work that we publish. This form provides structure for consistency and transparency in reporting. For further information on Nature Portfolio policies, see our Editorial Policies and the Editorial Policy Checklist.

Statistics

For all statistical analyses, confirm that the following items are present in the figure legend, table legend, main text, or Methods section.

n/a	Confirmed	
☐	☒	The exact sample size (*n*) for each experimental group/condition, given as a discrete number and unit of measurement
☐	☒	A statement on whether measurements were taken from distinct samples or whether the same sample was measured repeatedly
☐	☒	The statistical test(s) used AND whether they are one- or two-sided<br>*Only common tests should be described solely by name; describe more complex techniques in the Methods section.*
☒	☐	A description of all covariates tested
☐	☒	A description of any assumptions or corrections, such as tests of normality and adjustment for multiple comparisons
☐	☒	A full description of the statistical parameters including central tendency (e.g. means) or other basic estimates (e.g. regression coefficient) AND variation (e.g. standard deviation) or associated estimates of uncertainty (e.g. confidence intervals)
☐	☒	For null hypothesis testing, the test statistic (e.g. *F*, *t*, *r*) with confidence intervals, effect sizes, degrees of freedom and *P* value noted<br>*Give P values as exact values whenever suitable.*
☒	☐	For Bayesian analysis, information on the choice of priors and Markov chain Monte Carlo settings
☒	☐	For hierarchical and complex designs, identification of the appropriate level for tests and full reporting of outcomes
☒	☐	Estimates of effect sizes (e.g. Cohen's *d*, Pearson's *r*), indicating how they were calculated

Our web collection on statistics for biologists contains articles on many of the points above.

Software and code

Policy information about availability of computer code

Data collection	All the programs used for collecting data are reported in the Methods section of the paper and/or in the figures.
Data analysis	All the programs used for data analysis are reported in the Methods section of the paper and/or in the figures.

For manuscripts utilizing custom algorithms or software that are central to the research but not yet described in published literature, software must be made available to editors and reviewers. We strongly encourage code deposition in a community repository (e.g. GitHub). See the Nature Portfolio guidelines for submitting code & software for further information.

Data

Policy information about availability of data

All manuscripts must include a data availability statement. This statement should provide the following information, where applicable:

- Accession codes, unique identifiers, or web links for publicly available datasets
- A description of any restrictions on data availability
- For clinical datasets or third party data, please ensure that the statement adheres to our policy

All raw sequencing data generated furing the course of this project will be made publicly available in an online repository (Genbank) before publication. Theassembled transcriptomes will be additionally made available via the PlanMine website (https://planmine.mpibpc.mpg.de/planmine/begin.do).

Research involving human participants, their data, or biological material

Policy information about studies with human participants or human data. See also policy information about sex, gender (identity/presentation), and sexual orientation and race, ethnicity and racism.

Reporting on sex and gender	N.A.
Reporting on race, ethnicity, or other socially relevant groupings	N.A.
Population characteristics	N.A.
Recruitment	N.A.
Ethics oversight	N.A.

Note that full information on the approval of the study protocol must also be provided in the manuscript.

Field-specific reporting

Please select the one below that is the best fit for your research. If you are not sure, read the appropriate sections before making your selection.

☐ Life sciences ☐ Behavioural & social sciences ☒ Ecological, evolutionary & environmental sciences

For a reference copy of the document with all sections, see nature.com/documents/nr-reporting-summary-flat.pdf

Ecological, evolutionary & environmental sciences study design

All studies must disclose on these points even when the disclosure is negative.

Study description	Our study systematically explores the gain and loss of head regeneration in planarians by means of a live collection of more than 40 planarian species. Our experimental approach includes the quantification of species-specific head regeneration abilities, WISH (whole mount in situ hybridization), gene function analysis by RNAi, histology and quantitative Western Blotting to measure protein abundance. Experiments included a minimum of three individuals/species, depending on experimental requirements and animal availability. The association between regeneration ability, reproduction mode and ß-CATENIN-1 abundance was tested using linear mixed-effects models.
Research sample	Our study involved a broad range of planarian species that represent the major branches of planarian phylogeny. Our species collection was established by dedicated field sampling in known hotspots of planarian diversity. Between 10 to 100 individuals/species were collected at a field location and subsequently established as a laboratory population. Our study assumes those individuals and their offspring as representatives of the species. The transcriptome assemblies are based on RNA extracted from 3 to 6 individuals of field-collected populations. To measure head-regeneration abilities, we applied the standardised amputation-regeneration paradigms described in the text. All stainings were performed on individuals derived from progeny of field-collected populations except for S. mediterranea, of which we maintain clonal laboratory populations. Planarians are not subject to ethics approval in the EU.
Sampling strategy	All laboratory experiments were conducted with sufficient sample sizes for the specific purposes of the experiment, in line with the standards of the planarian research community.
Data collection	All data collection contributors are among the authors of the paper (please see the author's contribution statement). The data collection procedure for specific experiments is detailed in the Methods section.
Timing and spatial scale	Field collections were performed mainly before 2015. Experiments on the laboratory strains derived from field-collected specimens were performed between 2014-2021.
Data exclusions	In the case of Quantitative Western blotting (Fig. 4a and b, 5d, 5h to k, 6b, 6h), some replicates were excluded for obvious technical failures (e.g., insufficient band resolution due to DNA contamination). In the histology quantifications of yolk gland cross-sectional areas in Figures 6e and 6f, some replicates were excluded due to the absence of yolk glands in specific specimens. For this reason, we state in the text that " This tendency was statistically significant despite substantial inter-animal variations in the yolk content in some species (e.g., P. tenuis, P. torva or Camerata robusta) (Fig. 6f), which may reflect non-synchronous reproduction cycles under our laboratory culture conditions".
Reproducibility	The number of technical and biological replicates is clearly stated in the text.
Randomization	Specimens were selected randomly from laboratory populations. Where necessary, sexually mature individuals were preselected based on the presence or absence of a gonopore.

Blinding	Blinding was not necessary due to the use of either unambiguous assays (e.g., presence-absence of eyes as an indicator of head regeneration) or quantitative assays (e.g., fluorescence quantification of WB band intensities).

Did the study involve field work? ☒ Yes ☐ No

Field work, collection and transport

Field conditions	We conducted field sampling in multiple localities worldwide (see map in Fig. 1a). Sampling was performed as described in the manuscript. Seasonal variations in species abundance were not part of this study and were not analysed.
Location	A map with the sampled locations is shown in Fig. 1a. More precise data will be provided via planmine.
Access & import/export	Our collection is compliant with the Nagoya agreement (ascertained in collaboration with the German Nagoya Hub). Our fieldwork generally involved local researchers that are more familiar with the local regulations (see author contributions). Field sampling in Australia was performed under the Australian permit to take wildlife for scientific purposes No: 12239. Field sampling in Brazil was performed under the Brazilian permit for sampling No:02947.
Disturbance	Our sampling approach is minimally invasive per se, since we manually collect a small number of animals from submerged stones or other objects. We further minimise environmental impact by returning stones to their original position in the river bed and by avoiding sensitive habitats altogether.

Reporting for specific materials, systems and methods

We require information from authors about some types of materials, experimental systems and methods used in many studies. Here, indicate whether each material, system or method listed is relevant to your study. If you are not sure if a list item applies to your research, read the appropriate section before selecting a response.

Materials & experimental systems

n/a	Involved in the study	
☐	☒	Antibodies
☒	☐	Eukaryotic cell lines
☒	☐	Palaeontology and archaeology
☐	☒	Animals and other organisms
☒	☐	Clinical data
☒	☐	Dual use research of concern
☒	☐	Plants

Methods

n/a	Involved in the study	
☒	☐	ChIP-seq
☒	☐	Flow cytometry
☒	☐	MRI-based neuroimaging

Antibodies

Antibodies used	This study involved five antibodies. The penta-His, H3 and H3P antibodies are commercial. Our anti-Smed-b-CATENIN-1 monoclonal anitbody clone G78 was described previously (publication reference provided in the manuscript). The methods used for producing and testing the custom-raised anti-FERRITIN antibody, clone EO95, are described in the manuscript. Both antibodies are available upon request from the corresponding authors.
Validation	The methods used for validating the clone EO95 are described in the manuscript and involved the loss of the band upon RNAi-mediated knock-down of the epitope.

Animals and other research organisms

Policy information about studies involving animals; ARRIVE guidelines recommended for reporting animal research, and Sex and Gender in Research

Laboratory animals	We used two laboratory strains of Schmidtea mediterranea: the clonal CIW4 strain (asexual) and the inbred strain S2 (sexual). Both are established laboratory lines that are studied in many laboratories worldwide. Additionally, we also use laboratory strain of Dugesia japonica and Dugesia tahitiense.
Wild animals	The field-collected species and collection techniques are detailed in the manuscript. Briefly, animals were shipped on wet ice in 50 ml falcon tubes, at a density of ~20 individuals per tube, with 45 ml of water from the collection locality. The combinatorial method detailed in the manuscript was used to establish suitable laboratory culture conditions. Experiments were mainly performed on species from which we obtained stable laboratory cultures.
Reporting on sex	Sex considerations generally do not apply, as the planarians used in this study are either hermaphrodites or asexual. The exceptions are Sabussowia dioica (species with separate sexes) and Hymanella retenuova (likely a sequential hermaphrodite). Sex was not taken

into account when producing transcriptomes for those species or for the head regeneration assay in H. retenuova due to the difficulty of sexing live animals. The degree of sexual maturity of hermaphrodites, which is relevant for some of our experiments, was assessed by the presence of a gonopore. Conversely, the absence of the gonopore was used to ascertain the asexuality of specific strains.

Field-collected samples	General culture parameters for field-collected species are described in the manuscript, as are typical fixation and lysis protocols.
Ethics oversight	N.A. -Planarians are not subject to ethical oversight regulations.

Note that full information on the approval of the study protocol must also be provided in the manuscript.
